# Hirshfeld atom refinement and dynamical refinement of hexagonal ice structure from electron diffraction data

**DOI:** 10.1107/S2052252524006808

**Published:** 2024-07-30

**Authors:** Michał Leszek Chodkiewicz, Barbara Olech, Kunal Kumar Jha, Paulina Maria Dominiak, Krzysztof Woźniak

**Affiliations:** a Biological and Chemical Research Centre, Department of Chemistry University of Warsaw Żwirki i Wigury 101 Warszawa Warszawa02-089 Poland; b Centre of New Technologies University of Warsaw S. Banacha 2c Warsaw02-097 Poland; Ben-Gurion University of the Negev, Israel

**Keywords:** Hirshfeld atom refinement, dynamical scattering effects, kinematical HAR, electron diffraction, hexagonal ice, dynamical refinement

## Abstract

Reaching beyond the commonly used spherical atomic electron density model allows one to greatly improve the accuracy of hydrogen atom structural param­eters derived from X-ray data. However, the effects of atomic asphericity are less explored for electron diffraction data. In this work, Hirshfeld atom refinement (HAR), a method that uses an accurate description of electron density by quantum mechanical calculation for a system of interest, was applied for the first time to the kinematical refinement of electron diffraction data. This approach was applied here to derive the structure of ordinary hexagonal ice (I_h_). The effect of introducing HAR is much less noticeable than in the case of X-ray refinement and it is largely overshadowed by dynamical scattering effects. It led to only a slight change in the O—H bond lengths (shortening by 0.01 Å) compared with the independent atom model (IAM). The average absolute differences in O—H bond lengths between the kinematical refinements and the reference neutron structure were much larger: 0.044 for IAM and 0.046 Å for HAR. The refinement results changed considerably when dynamical scattering effects were modelled – with extinction correction or with dynamical refinement. The latter led to an improvement of the O—H bond length accuracy to 0.021 Å on average (with IAM refinement). Though there is a potential for deriving more accurate structures using HAR for electron diffraction, modelling of dynamical scattering effects seems to be a necessary step to achieve this. However, at present there is no software to support both HAR and dynamical refinement.

## Abstract 2

Kinematical Hirshfeld atom refinement has been applied to electron diffraction data for the first time, but the effect of using an aspherical atom model is overshadowed by dynamical scattering effects. Dynamical independent atom model refinement leads to significantly improved structures, suggesting that dynamical refinement is also necessary to obtain the full advantage of using aspherical atom models.

## 1. Introduction

Ice is one of the most abundant materials on Earth, common also in many other celestial bodies of the solar system. It is also one of the most important and the most studied materials. The structure of ordinary hexagonal ice (I_h_) has long been established by X-ray diffraction (Barnes, 1929 ▸) which led to the determination of oxygen positions. Neutron diffraction measurements of heavy ice with powder (Wollan *et al.*, 1949 ▸) and single-crystal (Peterson & Levy, 1957 ▸) diffraction later allowed the determination of hydrogen atom positions. Neutron measurements revealed hydrogen atom disorder consistent with a model proposed by Pauling (1935 ▸), with two half-occupied hydrogen sites lying at lines joining neighbouring oxygen atoms. Further single-crystal neutron diffraction studies (Kuhs & Lehmann, 1981 ▸, 1983 ▸) have confirmed the unusually long O—H distances: about 1.004 Å when the harmonic model of atomic displacement was used [similar results from powder neutron diffraction were also obtained by Fortes *et al.* (2015) ▸]. The results from the study by Kuhs & Lehmann (1981 ▸, 1983 ▸) are used as a reference in this work since they are of very high precision (O—H bond length standard deviation not exceeding 0.6 mÅ). An electron density distribution study with X-ray diffraction (Goto *et al.*, 1990 ▸) included anharmonic treatment of atomic displacement for oxygen and hydrogen atom positions. Recently, the structure of ice has been studied with 3D electron diffraction (3D ED) (Martynowycz & Gonen, 2019 ▸). 3D ED is an umbrella term (see *e.g.* Gemmi *et al.*, 2019 ▸) for several techniques for collecting ED data in 3D reciprocal space. The technique allows for structure determination from even very small samples, but data interpretation is hampered by strong dynamical scattering effects. A refinement method dedicated to the treatment of the dynamical scattering effects in 3D ED was developed by Palatinus *et al.* (2015*a* ▸) which significantly improves the accuracy of structure parameters (Palatinus *et. al*, 2015 ▸*b*) compared with the kinematical refinement.

Reference neutron measurement values of the two non-equivalent O—H bond lengths in hexagonal ice are very similar: 1.0040 (6) and 1.0036 (3) Å. Both X-ray and ED-derived structures exhibit O—H bond lengths that are considerably different from the neutron measurement values. Short (below 0.9 Å), in the case of X-rays, and long in the case of 3D ED (1.05 and 1.13 Å), bond lengths may be at least partially attributed to the applied spherically symmetric models of atomic electron density (X-ray) and electrostatic potential (3D ED). Such a model [independent atom model (IAM)] is commonly employed in X-ray and electron crystallographic refinement. It neglects the effects of chemical bonding and interatomic interactions in the solid state. This results in poor structural parameters for the hydrogen atoms – covalent bonds to hydrogen are too short in the case of X-rays (by about 0.1 Å) and it is usually not possible to determine the anisotropic ADPs. In practice, there are two commonly used aspherical models which can be used for deriving (freely refining) the structural parameters of hydrogen: transferable aspherical atom model (TAAM) and Hirshfeld atom refinement (HAR).

TAAM is based on the assumption of atomic density transferability between atoms in similar chemical environments and uses the Hansen–Coppens multipole model (Hansen & Coppens, 1978 ▸) for the parameterization of atomic densities (Pichon-Pesme *et al.*, 1995 ▸). TAAM leads to much more accurate structural parameters than IAM, as shown previously for several databanks of atomic electron density parameters (Zarychta *et al.*, 2007 ▸; Domagała *et al.*, 2012 ▸; Nassour *et al.*, 2017 ▸; Dittrich *et al.*, 2004 ▸, 2013 ▸; Volkov *et al.*, 2007 ▸; Dominiak *et al.*, 2007 ▸; Bąk *et al.*, 2011 ▸; Jarzembska & Dominiak, 2012 ▸; Jha *et al.*, 2020 ▸).

HAR (Jayatilaka & Dittrich, 2008 ▸; Capelli *et al.*, 2014 ▸) uses electron densities calculated for the system of interest which makes it more computationally demanding than TAAM, but avoids assumptions related to transferability and takes into account intermolecular interactions which, in principle, make it more accurate than TAAM. Atomic densities in HAR are obtained by Hirshfeld partition (Hirshfeld, 1977 ▸) of the total electron density. Other partitions were also tested (Chodkiewicz *et al.*, 2020 ▸) but none were clearly best. Though most of the HAR refinements were performed using wavefunction calculations for isolated systems, it was also possible to use a periodic wavefunction model (Wall, 2016 ▸; Ruth *et al.*, 2022 ▸). HAR on X-ray diffraction data produced hydrogen bond lengths very similar to those obtained from neutron diffraction experiments (*e.g.* Capelli *et al.*, 2014 ▸; Woińska *et al.*, 2014 ▸, 2016 ▸; Fugel *et al.*, 2018 ▸; Sanjuan-Szklarz *et al.*, 2020 ▸; Ruth *et al.*, 2022 ▸); however, the difference could be quite significant in the case of hydrogen bonded to heavy elements (Woińska *et al.*, 2021 ▸).

TAAM had not yet been used for an ice structure study. HAR had been used only once to obtain an ice VI structure from X-ray data (Chodkiewicz *et al.*, 2022 ▸) which lead to a much more accurate structure than that from IAM refinement.

Although the advantages of these models are pretty well established for X-ray diffraction, the effect of atomic asphericity in ED is much less explored. The introduction of 3D electron crystallography (Kolb *et al.*, 2007 ▸) started a period of rapid development of ED techniques applicable to crystal structure determination.

Electrostatic potentials determine electron scattering, which leads to rather different scattering factors for neutral and charged atoms (see *e.g.* Saha *et al.*, 2022 ▸). Application of a partial charge model (Yonekura & Maki-Yonekura, 2016 ▸) allowed for improved fitting statistics.

While both TAAM and HAR form factors are based on atomic electron densities with partial atomic charges, they also model the asphericity of the atomic charge distribution. This feature was already modelled 20 years ago by Zhong *et al.* (2002 ▸), who used the superposition of potentials calculated for small fragments to model electrostatic potentials in a larger system. They observed significant differences in scattering factors when compared with free atom superposition, especially for low-resolution reflections. Aspherical scattering factors for electrons were parameterized by Zheng *et al.* (2009 ▸) by parameterizing scattering from *p* and *d* orbitals; however, its practical application requires knowledge of the orbital orientation and occupancy.

A refinement using aspherical atom form factors against electron scattering data has been performed with TAAM (Gruza *et al.*, 2020 ▸). Data for a small molecule (carbamazepine) have been used. Refinements within kinematical approximation were performed against both experimental and theoretically modelled data (to avoid effects originating from dynamical scattering). The conclusions from this work were drawn mostly on the basis of model data since the experimental data gave much higher discrepancies in bond lengths and ADPs for both TAAM and IAM, and some trends visible for the model data were probably obscured by experimental errors or dynamical scattering effects. Overall, in the case of the refinement against model data, a better agreement between the structural parameters and better refinement statistics have been observed for TAAM (compared with IAM), with significantly better ADPs for non-hydrogen atoms and slightly better hydrogen atom positions (which were already quite good with IAM, the average root mean square deviation (RMSD) was 0.02 versus 0.01 Å for TAAM). RMSDs for *X*—H bond lengths from refinement against experimental data were similar for TAAM and IAM refinements: 0.075 Å for TAAM and 0.078 Å for IAM. TAAM was also used for refinement against experimental ED data for β-glycine (Jha *et al.*, 2021 ▸). It was shown to give better refinement statistics and more reasonable *X*—H bond lengths than IAM.

In this work, we test HAR for 3D ED. The techniques are combined for the first time to assess the effect of modelling the asphericity of the atomic electron densities and the corresponding electrostatic potentials by the HAR approach on the kinematical refinement of 3D ED data. The method is applied to hexagonal ice (I_h_). A combination of HAR and dynamical refinement would be better suited for such an analysis, but there is no software yet that enables both techniques. Therefore, dynamical scattering effects in this work are modelled only in the case of IAM.

## 2. Measurements

Hexagonal ice crystals eventually appear whenever we prepare grids for data collection at the temperature of liquid nitro­gen by absorbing moisture from the surroundings. A glow discharged lacey carbon 200 mesh Cu grid was used for data collection. MicroED samples for ice VI crystals were prepared by directly applying the grid to liquid nitro­gen. A Thermo Fisher Scientific Glacios cryo transmission electron microscope (TEM) equipped with a field emission gun and operated at 200 kV and −192°C was used for data collection. The microscope was equipped with a Thermo Fisher Scientific CETA-D detector, an autoloader with a twelve-grid holder and *EPU-D* software for automated data collection. A 50 µm condenser aperture, spot size 11 and gun lens 8 were set and the diffraction datasets were collected under the parallel illumination condition with a very low dose. The crystal was continuously rotated from −55 to +55° with a tilt speed of 1° s^−1^ and frames were recorded after each rotation of 0.5°. The single frame exposure time was 0.5 s. A total of 219 image frames were collected. Data reduction was carried out in *CrysAlis* (Rigaku Corporation). In total, 54 virtual frames were made. Data collection details are given in Table S1 of the supporting information. Raw diffraction images and associated files documenting data reduction various refinement steps are available online (Chodkiewicz *et al.*, 2024 ▸) at https://doi.org/10.18150/IVF5BA (Repository for Open Data, Interdisciplinary Centre for Mathematical and Computational Modelling, University of Warsaw, Warsaw, Poland).

## 3. Refinements

### 3.1. Kinematical refinements

#### 3.1.1. In *Olex2*

For both HAR and IAM, three refinements were performed: one with all the reflections, one with some of the reflections omitted (presumably those which deviate most from experimental values, discussed in Section 4), and one with the extinction parameter refined and all reflections included.

IAM refinement in *Olex2* (version 1.5; Dolomanov *et al.*, 2009 ▸) was performed without *I*/σ(*I*) cut-off and using the parameterization by Peng (1999 ▸).

In the case of HAR, the electron atomic form factors *f_e_*(**s**) were calculated from the X-ray form factors *f_x_*(**s**) using the Mott−Bethe formula:
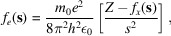
where *m*_0_ is the electron rest mass, ε_0_ is the permitivity under vacuum and *Z* is the atomic number.

Since the ice I_h_ structure is disordered, calculation of atomic form factors for HAR required incorporation of electron densities from multiple local conformations which could occur in real crystals. Atomic densities obtained with Hirshfeld partition of the total electron density were then averaged over the conformations. We have used clusters of five water molecules (a ‘central’ water molecule and its four nearest neighbours) to generate such local conformations. There are two different conformations of the central molecule: (1) containing H1 and H2; and (2) containing two H1 atoms, in addition each of the four neighbours of the central molecule has three possible conformations. Conformations of the neighbours were not modelled, instead one of them was assumed to provide good representation of the interaction between the central molecule and its neighbours (see Fig. S1). Other possible representations would differ in the position of the hydrogen atom which is not directly involved in the hydrogen bond to the central molecule and, as a consequence, probably only had a minor influence on modelling the effects of the intermolecular interactions. Two possible configurations of water molecules were used in the calculations.

A second-order Møller–Plesset perturbation theory (MP2) calculation with a *cc-pVTZ* basis set was used for the calculation of the electron densities. Quantum mechanical calculations were performed with *Gaussian* (Frisch *et al.*, 2016 ▸). Details of the HAR procedure are given in the supporting information.

HAR was performed using a locally modified version of *Olex2* (Dolomanov *et al.*, 2009 ▸). A program based on a development version of the *DiSCaMB* library (Chodkiewicz *et al.*, 2018 ▸) was used to generate files with atomic form factors in tsc format (Kleemiss *et al.*, 2021 ▸; Midgley *et al.*, 2019 ▸). Such files are then imported into *Olex2* and used in the refinement conducted with *olex2.refine*.

#### 3.1.2. In *Jana2020*

There were two kinematical IAM refinements performed in *Jana2020* (version 1.3.36; Petříček *et al.*, 2023 ▸): one with optimization of the extinction parameter and one without. The refinements were performed without using an *I*/σ(*I*) cut-off and with electron form factor parameterization from *International Tables of Crystallography* (Vol. C, ch. 4.3.1.1; Colliex *et al.*, 2006 ▸).

### 3.2. Dynamical IAM refinement

Similar to the kinematical refinement in *Jana2020*, no *I*/σ(*I*) cut-off was applied and the same source of form factors was used. The structure obtained from IAM refinement in *Olex2* was used as a starting point. All reflections with the maximal relative excitation error (RSg) equal to 0.4 were included in the refinement. The number of merged frames was set to six and the step between frames was set to four to obtain virtual frames. A sigma-weighting scheme was applied. Including the dynamical scattering description allowed the inclusion of all strong reflections in the refinement. *R*_1_ was calculated for the reflections with *I* > 3σ(*I*).

## 4. Results and discussion

The results of the refinements are reported in Table 1 ▸ and additional information is provided in Table S2. *R*_1_ values for refinements with *Jana2020* are reported for reflections with *I* > 3σ(*I*) and for *Olex2* refinements for *I* > 2σ(*I*).

### 4.1. Kinematical refinements

**Figure 1:**
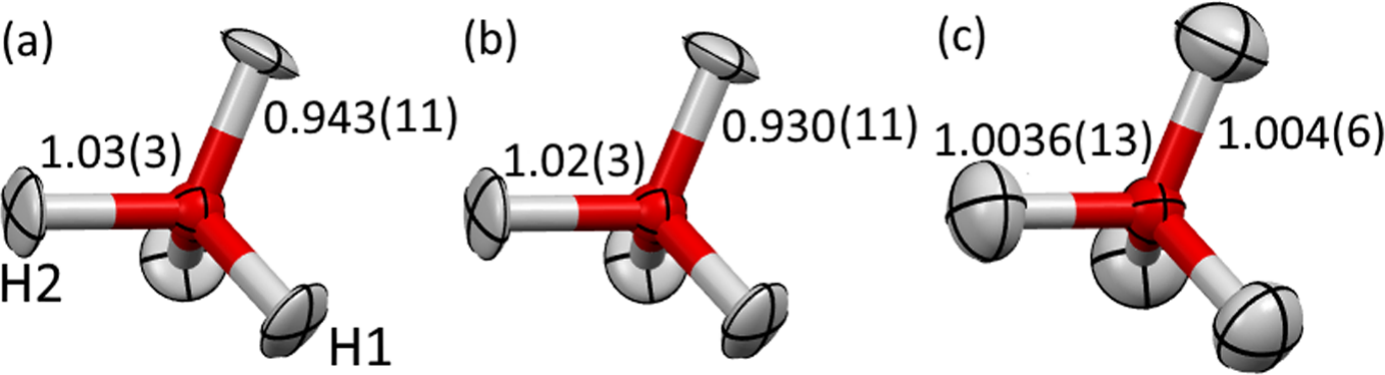
Ice structure from kinematical (*a*) IAM and (*b*) HAR refinement using all reflections and without extinction included, and (*c*) the neutron measurement.

While HAR gives a slightly better *R*_1_ agreement factor than IAM (19.34 versus 21.06%) and slightly cleaner but very similar residual density maps (Fig. S2), both kinematical HAR and IAM refinement lead to quite similar structures (see Fig. 1 ▸). The average absolute difference in O—H bond lengths [compared with the neutron structure (Kuhs & Lehmann, 1981 ▸) at 60 K] is 0.044 and 0.046 Å for IAM and HAR, respectively. Bonds in structures from HAR are slightly shorter (by about 0.01 Å) than those from IAM, which is an expected effect; similar differences were obtained from IAM and TAAM refinements against theoretical data (Gruza *et al.*, 2020 ▸). One of the O—H bonds is shorter than the other by as much as 0.09 Å, whereas they are almost the same [1.004 (6) and 1.0036 (13) Å] in the case of neutron data. A similar feature can be observed in the case of a 3D ED derived structure (Martynowycz & Gonen, 2019 ▸) (1.13 and 1.05 Å). In this case, the bond lengths were much larger than from the IAM refinement reported here [1.03 (3) and 0.943 (11) Å]. Certainly the effect of HAR on bond length (0.01 Å) was very small in comparison with the observed inaccuracies.

**Figure 2:**
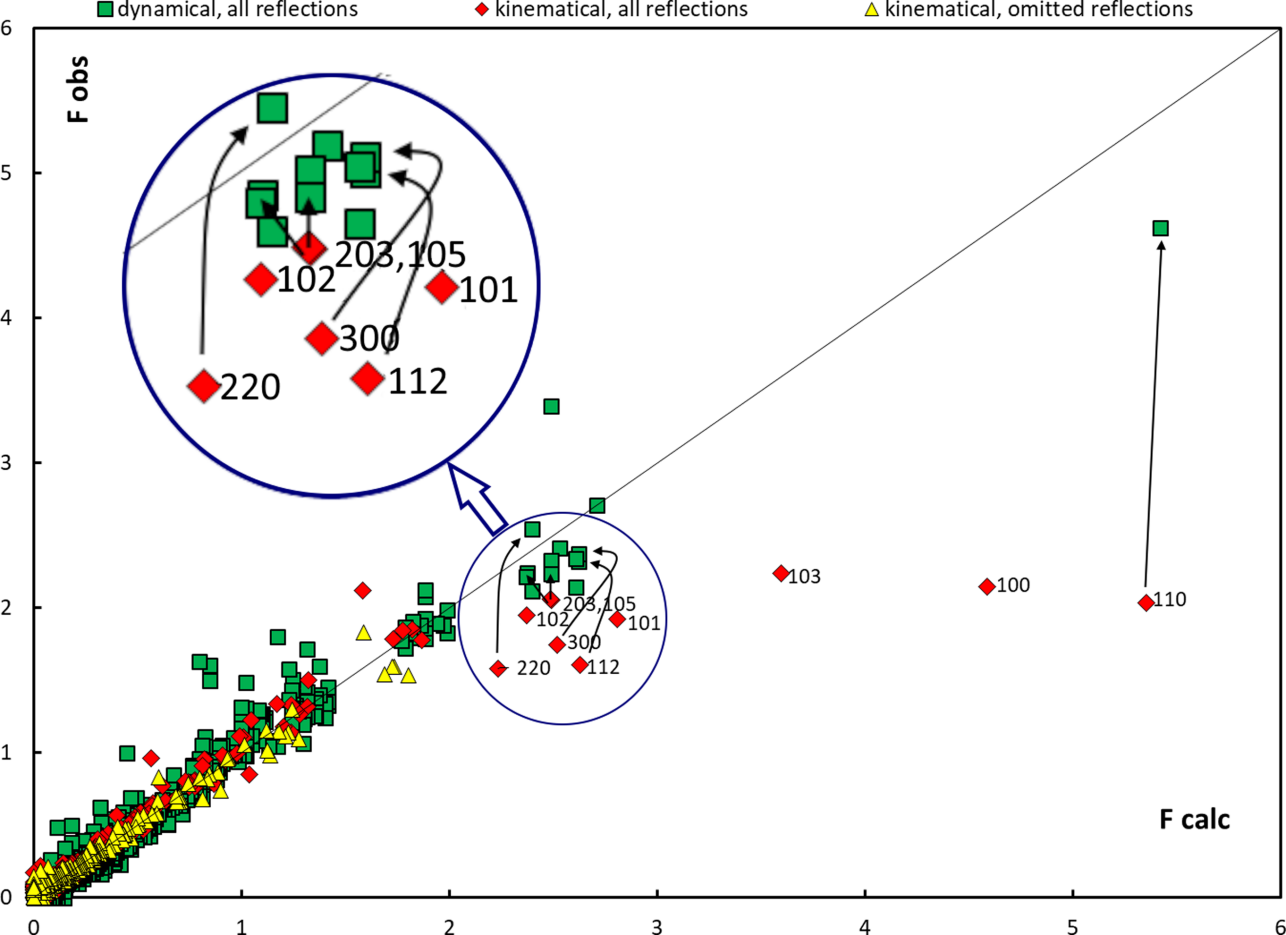
Observed versus calculated structure factor amplitudes for dynamical refinement (green squares) and kinematical IAM refinement (in *Olex2*) with all reflections included (red diamonds) and with some omitted (yellow triangles); the omitted reflections are labelled (a list is provided in the supporting information). Arrows point from the labelled reflections to the corresponding reflections in dynamical refinement, a missing arrow indicates a lack of the reflection in the dynamical refinement (some of them were automatically treated as outliers).

Equivalent isotropic atomic displacement parameters (*U*_eq_) for hydrogen atoms from IAM and HAR are much smaller than the corresponding neutron diffraction values (on average 0.018 versus 0.027 Å^2^) and also smaller but to a lesser extent for oxygen atoms (0.012 versus 0.014 Å^2^). A comparison of calculated and experimental structure factors revealed (Fig. 2 ▸, red diamonds) that some intense reflections had significantly lower intensities than the model examples, which might be related to dynamical scattering effects. On average, dynamical scattering increases the intensity of weaker reflections, whereas the strong reflections become less intense. Similarly, decreasing the ADPs would make high-angle intensities larger. As a result, the dynamical scattering effect can be partially modelled by artificial decreasing of ADPs and this might be the reason why the ADPs are too small.

Refinements with some of the strongest reflections omitted (see Fig. 2 ▸, the labelled reflections and the supporting information) and refinements with extinction optimization (as a way to model effects of dynamical scattering) were also performed (see Table 1 ▸ for a summary of all the refinements). The reflections with the highest difference between observed and calculated intensities were chosen manually based on visual inspection. They lead to an increase of the ADPs close to the neutron values (on average 0.025 Å^2^ for omitted reflections, 0.0275 Å^2^ for refinement with extinction included and 0.027 Å^2^ for neutron measurements). Yet the problem with bond lengths remained at a similar scale.

In addition to kinematical refinements with *Olex2*, kinematical refinements with *Jana2020* were performed as they were more appropriate for comparison with dynamical refinements also performed with *Jana2020*. Contrary to *Olex2*, *Jana2020* does not use a *SHELX*-type weighting scheme. Such a scheme was used in the refinements described so far. In addition, *Olex2* refinements were based on |*F*|^2^ while *Jana2020* on |*F*|. O—H bond lengths from kinematical refinement with *Jana2020* were longer than those from *Olex2*, especially O—H2 (1.088 vs 1.030 Å). Hydrogen atom ADPs were refined isotropically, otherwise non-positive definite ADPs would appear for one of the hydrogen atoms.

### 4.2. Dynamical IAM refinement

Dynamical IAM refinement lowered *R* factors, *R*_1_ decreased by more than 4.75 percentage points relative to kinematical IAM refinement. However, during the refinement procedure, some reflections were automatically omitted (treated as outliers), namely 100, 101, 102 and 103. They were included in the kinematical refinement and were far from being well reproduced (see Fig. 2 ▸). The large discrepancy between the model and observed intensities for other strong reflections disappeared (Fig. 2 ▸). The bond lengths also significantly improved, *i.e.* the average absolute difference from reference neutron values dropped from 0.064 to 0.021 Å. However, it was still not possible to refine all hydrogen atoms with anisotropic ADPs.

### 4.3. Discussion

#### 4.3.1. Observations from previous refinements

An effect of atomic asphericity on the hydrogen atom parameters was quite large in the case of X-ray structure refinement. It may have also lead to a significant lowering of the agreement factors (*R* factors). For discussion of the current results, it was convenient to focus on the accuracy of the lengths of covalent bonds to hydrogen (*X*—H), since the other parameters – ADPs and *R* factors – can be strongly influenced by dynamical scattering (Palatinus *et al.*, 2015*a* ▸; Klar *et al.*, 2023 ▸). Inclusion of the asphericity of atomic electron densities in the refinement models usually led to substantial elongation of the *X*—H bonds (usually by more than 0.1 Å) in the case of X-ray structure refinement, but it is predicted to be modest in the case of 3D ED. IAM refinement against quantum mechanically simulated 3D ED intensities for carbamazepine (Gruza *et al.*, 2020 ▸) led to slightly long *X*—H bonds [0.024 Å on average for 0.83 Å resolution and only by 0.006 Å for 0.6 Å resolution (RMSDs of 0.024 and 0.012 Å, respectively)]. Experimental verification of this prediction, however, was hindered by the presence of dynamical scattering effects in 3D ED which is typically not accounted for. The differences from refinement against simulated data were far smaller than those from refinements against experimental data for the same system. Using the neutron structure as a reference gave an RMSD of 0.078 Å for IAM and nearly the same (0.075 Å) for TAAM *X*—H bond lengths. Even higher differences occurred in the case of β-glycine (Jha *et al.*, 2021 ▸). While there was no reference structure to use for comparison in this case, some of the *X*—H bond lengths were clearly too large, *e.g.* for C—H it reached 1.32 (8) Å for IAM and 1.17 (5) Å for TAAM whereas the average from the neutron measurements was 1.097 Å (Allen & Bruno, 2010 ▸).

#### 4.3.2. Discussion of the current results

In the current study, *X*—H bonds from IAM are on average only 0.01 Å longer than those from HAR. The differences between IAM and aspherical model values were larger in the previous studies – the bonds obtained with TAAM were on average 0.036 Å longer for carbamazepine (Gruza *et al.*, 2020 ▸; 3D ED experimental data) and 0.055 Å longer for β-glycine (Jha *et al.*, 2021 ▸) in comparison with the IAM values. In the case of ice, these differences are of the order of standard uncertainties (0.01 and 0.03 Å in the case of HAR) for bond lengths, but the trend seemed to be quite consistent; all *X*—H bonds from IAM were longer in the case of ice and glycine.

Although we were able to observe the asphericity-related trends in bond lengths, which were predicted using simulated data, the related effects were relatively small. They were much smaller than the effect of modelling dynamical scattering effects with the refinement of extinction parameter or changes related to using different least squares software (probably as a result of using a different weighting scheme and/or different objective function – refinement on |*F*| in *Jana2020* and on |*F*|^2^ in *Olex2*). Dynamical refinement largely improved the refinement statistics and accuracy of the structure. At present, it is not possible to perform HAR with dynamical refinement and it is also difficult to fully assess its influence on structure refinement with ED data. While there is no additional theoretical development needed for implementing dynamical HAR, no software (that we are aware of) currently supports such refinement.

## 5. Conclusions

Kinematical HAR against 3D ED data has been performed for the first time. HAR brings clear improvement in the case of X-ray diffraction, yet in the case studied here – ice I_h_ – its advantages were still overshadowed by errors introduced by a lack of modelling of the dynamical scattering not being accounted for.

In the case of the investigated structure, it was possible to freely refine hydrogen atom positions and anisotropic ADPs for both HAR and IAM in kinematical refinement.

The average absolute differences in O—H bond lengths between the reference neutron structure and both for HAR and for IAM were between 0.035 and 0.053 Å, on average 0.044 Å for IAM and 0.046 Å for HAR. In the case of X-ray refinements, such differences for IAM are usually one order of magnitude larger than for HAR, here they are very similar. The two O—H bond lengths from the ED experiment differed quite considerably (by about 0.06–0.07 Å), yet they were almost identical in the case of neutron data. It is expected that application of aspherical atomic electron density models would lead to shorter *X*—H bonds and indeed bonds from HAR were shorter than those from IAM, but only by 0.01 Å on average. The effects of using HAR instead of IAM in kinematical refinements were quite small in comparison with the inaccuracies of the structures obtained (the 0.01 Å difference in bond lengths versus at least 0.035 Å error in bond lengths). Dynamical refinement was only possible in the case of IAM and considerably improved the resulting structure, the bond lengths became closer to neutron values (0.021 Å on average) and the large discrepancies between observed and calculated intensities were radically reduced.

HAR may potentially produce better structural models from 3D ED than IAM; however, it did not show any advantage over IAM in the structure studied and the differences between HAR- and IAM-derived structures were relatively small. Modelling of the dynamical scattering effects seems to be necessary to take advantage of a more advanced HAR model.

## Supplementary Material

Crystal structure: contains datablock(s) Jana2020_IAM, JANA2020_IAM_dynamic, Jana2020_IAM_exti, HAR, har_exti, har_omit, Olex_IAM, Olex_iam_exti, Olex_iam_omit. DOI: 10.1107/S2052252524006808/vq5006sup1.cif

Supporting tables and figures. DOI: 10.1107/S2052252524006808/vq5006sup2.pdf

CCDC references: 2370012, 2370013, 2370014, 2370015, 2370016, 2370017, 2370018, 2370019, 2370020

## Figures and Tables

**Table 1:** Summary of refinements *R*_1_ agreement factor (%), calculated for *I* > *n*σ(*I*) (*n* = 3 for *Jana2020*, *n* = 2 for *Olex2*). O—H bond lengths (Å) and average absolute difference of the bond lengths (Å) from electron and neutron diffraction experiments. Refinements tagged with ‘exti’ involve extinction refinement and ‘omit’ indicates that some of strongest reflections were omitted [see Fig. 2 ▸(*a*) and the supporting information].

Model/measurement	*R*1(*I* > *n*σ[*I*)]	Bond length		〈|Δ*R*|〉
		O—H1	O—H2	
Refinements with *Olex2* on |*F*|^2^				
IAM kinematical	22.09	0.943 (11)	1.03 (3)	0.044
IAM kinematical exti	12.34	0.951 (8)	1.03 (2)	0.039
IAM kinematical omit	12.12	0.935 (12)	1.00 (2)	0.035
HAR kinematical omit	10.62	0.911 (13)	0.99 (2)	0.053
HAR kinematical	19.34	0.930 (11)	1.02 (3)	0.046
HAR kinematical exti	11.43	0.933 (9)	1.01 (2)	0.041

Refinements with *Jana2020* on |*F*|				
IAM kinematical	20.96	0.96 (3)	1.09 (8)	0.064
IAM kinematical exti	14.34	0.885 (16)	1.04 (5)	0.076
IAM dynamical	10.21	0.998 (8)	1.037 (18)	0.021
Neutron†	–	1.0040 (6)	1.0036 (13)	–

† Kuhs & Lehmann (1981 ▸).

## Data Availability

The authors confirm that the data supporting the findings of this study are available within the article and its supporting information.
