# Supplementary material for: Hirshfeld atom refinement and dynamical refinement of hexagonal ice structure from electron diffraction data
Source: IUCrJ. 2024 Jul 30;11(Pt 5):730–6. doi: 10.1107/S2052252524006808 (PMC11364029; doi:10.1107/S2052252524006808)
Supplement: Supplementary file 2 [file m-11-00730-sup2.pdf]

**IUCrJ**

**Volume 11 (2024)**

**Supporting information for article:**

## Hirshfeld Atom Refinement and Dynamical Refinement of Hexagonal Ice Structure from Electron Diffraction Data

**Michał Leszek Chodkiewicz, Barbara Olech, Kunal Kumar Jha, Paulina Maria Dominiak and Krzysztof Woźniak**

**Table S1** Supporting information Data collection details.

| | |
|---|---|
| T(K) | 81.15 K |
| Space group | P $6_3$/mmc |
| a=b, c (Å) | 4.3815(13), 7.183(4) |
| V(Å$^3$) | 119.42(8) |
| No. of measured reflections, unique and observed reflections ($I > 2\sigma(I)$, or $I > 3\sigma(I)$) kinematical refinement | 2005, 265, 215, 199 |
| No. of measured reflections, unique and observed reflections [$I > 3\sigma(I)$], dynamical refinement | 5180, 1106, 623 |
| No. of parameters | 13 |
| $(\sin \theta/\lambda)_{max}$(Å$^{-1}$) | 1.1124 |
| Data completeness | 0.895 |
| $R_{int}$ | 7.24% |

**List of the strong reflections which were omitted in some of the refinements.**

1 1 0, 1 0 0, 1 0 3, 1 1 2, 3 0 0, 1 0 1, 2 2 0, 1 0 2, 2 0 3, 1 0 5

**Table S2** Refinement statistics. R1 agreement factors for all reflections used in refinement and for reflections with I>3σ(I) (for Jana2020) and I>2σ(I) for Olex2. wR agreement factors as $wR_1$ for Jana 2020 and $wR_2$ for Olex2. Shelx type weighting scheme $w = 1/(\sigma^2(F_o^2)+(aP)^2+bP)$ parameters a and b are provided for Olex2 refinements only since Jana2020 does not use such weighting scheme. GOF stands for goodness of fit. Refinements tagged with ‘exti’ involve extinction refinement, ‘omit’indicates that some of strongest reflections were omitted (listed above), ‘dynamical’ indicates dynamical refinement.

| refinement | R1 | | wR | | Weighting scheme | | GOF |
|---|---|---|---|---|---|---|---|
| | I>nσ(I) | all | gt | ref | a | b | |
| Refinements with Olex2 on $\|F\|^2$ | | | | | | | |
| IAM | 21.06 | 22.09 | 38.82 | 40.04 | 0.2 | 0 | 1.6241 |
| IAM exti | 10.89 | 12.34 | 32.72 | 34.04 | 0.2 | 0 | 1.3472 |
| IAM omit | 10.31 | 12.12 | 27.29 | 28.76 | 0.158 | 0.002 | 1.0107 |
| HAR omit | 10.62 | 12.46 | 28.12 | 29.55 | 0.157 | 0.002 | 1.0522 |
| HAR | 19.34 | 20.44 | 38.69 | 37.60 | 0.171 | 0 | 1.7647 |
| HAR exti | 11.43 | 12.88 | 33.63 | 34.91 | 0.2 | 0 | 1.3836 |
| Refinements with Jana2020 on $\|F\|$ | | | | | | | |
| IAM | 20.96 | 23.03 | 23.72 | 23.92 | | | 9.2439 |
| IAM exti | 14.34 | 16.57 | 16.29 | 16.57 | | | 6.4507 |
| IAM dynamical | 10.21 | 12.82 | 12.31 | 12.87 | | | 2.5372 |

**The water clusters used in wave function calculation in HAR.**

The atoms used in the cluster construction (atom and symmetry code):

Cluster 1: O1 x,y,z; H1 x,y,z; H1 1-y,1+x-y,z; O1 1-y,1-x,1/2-z; H1 1-y,1-x,1/2-z; H2 1-y,1-x,1/2-z; O1 1+x-y,1-y,1-z; H2 1+x-y,1-y,1-z; H1 y,x,1-z; O1 1+x-y,2-y,1-z; H2 1+x-y,2-y,1-z; H1 y,1+x,1-z;

O1 x-y,1-y,1-z; H2 x-y,1-y,1-z; H1 -1+y,x,1-z

Cluster 2: O1 x,y,z; H1 x,y,z; H2 x,y,z; O1 1-y,1-x,1/2-z; H1 1-y,1-x,1/2-z; H1 -x+y,y,1/2-z;

O1 1+x-y,1-y,1-z; H1 1+x-y,1-y,1-z; H1 1-x,-x+y,1-z; O1 1+x-y,2-y,1-z; H1 1-x,1-x+y,1-z;

H1 y,1+x,1-z; O1 x-y,1-y,1-z; H1 x-y,1-y,1-z; H1 -1+y,x,1-z

Their coordinates (from last cycle of HAR with all reflections used and no extinction modeling):

Cluster 1:

| | | | |
|---|---|---|---|
| O | 0.000000 | 2.529654 | 3.144831 |
| H | -0.758283 | 2.091860 | 3.457001 |
| H | 0.758283 | 2.091860 | 3.457001 |
| O | 0.000000 | 2.529654 | 0.446635 |
| H | 0.000000 | 3.405244 | 0.134464 |
| H | 0.000000 | 2.529654 | 1.467401 |
| O | 2.190745 | 1.264827 | 4.038100 |
| H | 2.190745 | 1.264827 | 5.058866 |
| H | 2.190745 | 0.389238 | 3.725930 |
| O | 0.000000 | 5.059309 | 4.038100 |
| H | 0.000000 | 5.059309 | 5.058866 |
| H | 0.000000 | 4.183720 | 3.725930 |
| O | -2.190745 | 1.264827 | 4.038100 |
| H | -2.190745 | 1.264827 | 5.058866 |
| H | -2.190745 | 0.389238 | 3.725930 |

Cluster 2:

| | | | |
|---|---|---|---|
| O | 0.000000 | 2.529654 | 3.144831 |
| H | -0.758283 | 2.091860 | 3.457001 |
| H | 0.000000 | 2.529654 | 2.124065 |
| O | 0.000000 | 2.529654 | 0.446635 |
| H | 0.000000 | 3.405244 | 0.134464 |
| H | 0.758283 | 2.091860 | 0.134464 |
| O | 2.190745 | 1.264827 | 4.038100 |
| H | 1.432462 | 1.702622 | 3.725930 |
| H | 2.949028 | 1.702622 | 3.725930 |
| O | 0.000000 | 5.059309 | 4.038100 |
| H | 0.758283 | 5.497104 | 3.725930 |
| H | 0.000000 | 4.183720 | 3.725930 |
| O | -2.190745 | 1.264827 | 4.038100 |
| H | -2.949028 | 1.702622 | 3.725930 |
| H | -2.190745 | 0.389238 | 3.725930 |

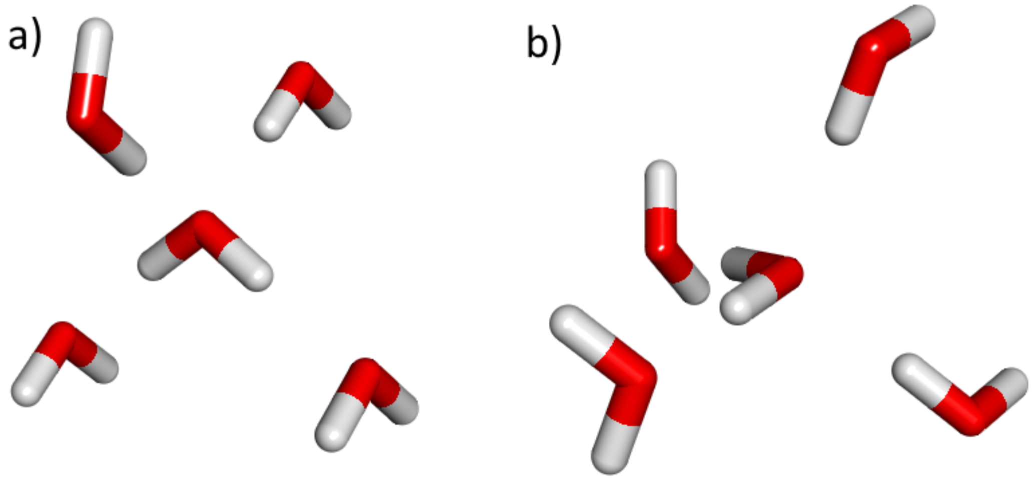


**Figure S1** Wave function calculations in HAR were computed for the two clusters of water molecules. Atomic electron densities of the atoms of the central water molecule were used in atomic form factors calculations.

**HAR implementation details**

The HAR procedure:

1. A locally modified version of Olex2 prints out structural parameters to text files with high accuracy.
2. Positional parameters are used for the calculation of the coordinates of atoms in the water clusters.
3. ORCA input files are printed out.
4. ORCA is called for wave function calculation, the wave function is saved in wfx format.
5. DiSCaMB library-based program reads in the wave function and calculates electron density on integration grid – with 590 point Lebedev-Laikov integration grid and 75 point Mura-Knowles radial integration grid (the same grid size is used for oxygen and hydrogen).
6. Atomic electron densities are calculated using Hirshfeld partition, isolated atom spherically averaged electron densities which are needed for Hirshfeld partition are precalculated at the radial grid with 0.001 Bohr step, the theory level matches the one used in the cluster wave function calculation. The values of spherical atom electron density are calculated using interpolation of those values.
7. X-ray atomic form factors (Fourier transforms of the atomic electron densities) are calculated via numerical integration of the atomic electron densities.

8. The X-ray atomic form factors are transformed into electron scattering form factors with Mott–Bethe formula and saved to tsc file.
9. The tsc file with the atomic form factors is read by Olex2 and used in refinement.
10. An utility program is called which check if convergence of HAR procedure is achieved by comparing the largest (parameter shift)/(σ(parameter)) with the threshold (set to 0.1). The parameter standard deviations are calculated using a variance-covariance matrix printed out by Olex2.
11. If the convergence is achieved the procedure stops, otherwise it is repeated (starting from the point 1).

**Difference density maps**

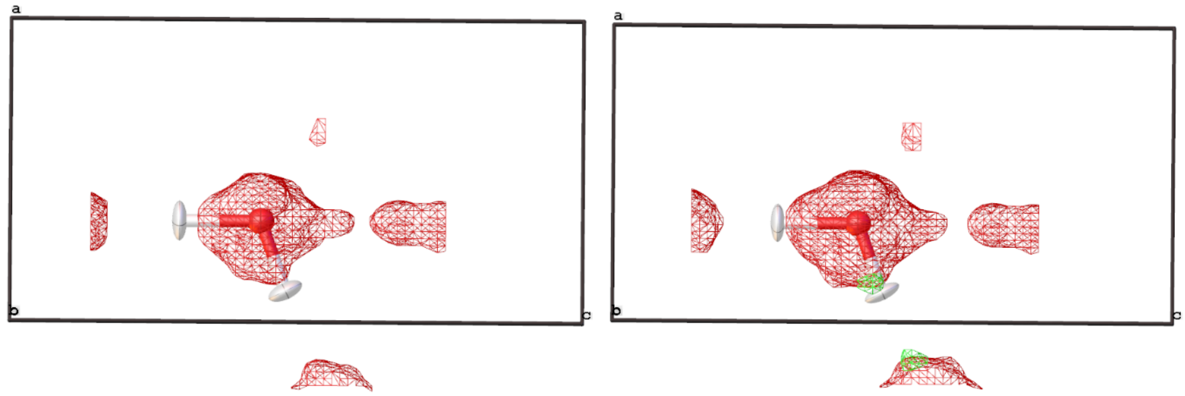


**Figure S2** Residual density maps for HAR with all reflections (left) and IAM with all reflections (right) at 0.2 Å$^{-2}$ level.
